# The Diagnostic Significance of Ultrasonographic Measurement of the Achilles Tendon Thickness for the Insertional Achilles Tendinopathy in Patients with Heel Pain

**DOI:** 10.3390/jcm10102165

**Published:** 2021-05-17

**Authors:** Du-Hwan Kim, Jae-Hyeong Choi, Chul-Hyun Park, Hee-Jin Park, Kyung-Jae Yoon, Yong-Taek Lee

**Affiliations:** 1 Department of Physical Medicine and Rehabilitation, College of Medicine, Chung-Ang University, Seoul 06973, Korea; ri-pheonix@hanmail.net; 2 Department of Physical and Rehabilitation Medicine, Kangbuk Samsung Hospital, Sungkyunkwan University School of Medicine, Seoul 03181, Korea; j810.choi@samsung.com (J.-H.C.); chulhyun.park@samsung.com (C.-H.P.); kjae.yoon@samsung.com (K.-J.Y.); 3 Department of Radiology, Kangbuk Samsung Hospital, Sungkyunkwan University School of Medicine, Seoul 03181, Korea; heejin6484@samsung.com

**Keywords:** insertional Achilles tendinopathy, plantar fasciitis, ultrasonography

## Abstract

No consensus exists concerning the diagnostic role or cutoff value of the Achilles tendon thickness on ultrasonography (US) for the diagnosis of insertional Achilles tendinopathy. This study sought to assess the diagnostic utility of US measurement of the thickness and echogenicity of the Achilles tendon for the insertional Achilles tendinopathy in patients with heel pain, and to compare the results with those of the plantar fascia for the plantar fasciitis. We conducted US examinations in consecutive patients who presented with unilateral or bilateral heel pain at the foot clinic of a single tertiary hospital from February 2016 to December 2020. Each US evaluation assessed the thickness and echogenicity of the insertion area of the Achilles tendon and plantar fascia. We retrospectively compared these parameters between patients with insertional Achilles tendinopathy or plantar fasciitis and normal controls and analyzed the diagnostic utility of these parameters. Based on clinical diagnosis, 44 feet were diagnosed with insertional Achilles tendinopathy, 109 feet were diagnosed with plantar fasciitis, and 32 feet were classified as normal. There was a significant difference in the thickness of the plantar fascia between the plantar fasciitis and normal control groups (*p* = 0.032). There was also a significant difference in the echogenicity of the plantar fascia between the plantar fasciitis and normal groups (*p* < 0.001). However, there was no significant difference in the thickness of the insertional area of the Achilles tendon between the insertional Achilles tendinopathy and normal groups (*p* = 0.132). There was a significant difference in the echogenicity of the insertional area of the Achilles tendon between the insertional Achilles tendinopathy and normal groups (*p* < 0.001). US measurement of the thickness of the insertional area of the Achilles tendon might not reflect the clinical status of insertional Achilles tendinopathy, unlike that of plantar fasciitis.

## 1. Introduction

Heel pain is a common clinical manifestation leading to a foot clinic visit [1]. The differential diagnosis of heel pain is extensive, including plantar fasciitis, plantar fibromatosis, heel pad syndrome, Achilles tendinopathy, and Haglund’s deformity with or without bursitis [1]. Among these conditions, plantar fasciitis and Achilles tendinopathy are the two most common diagnoses rendered for heel pain. The specific anatomical location of the pain helps with diagnosis. Both diseases are diagnosed through history-taking and physical examination. In patients with plantar fasciitis, the primary symptom is usually throbbing medial plantar heel pain that is worse with the first step after rest or in the morning and is often relieved by further ambulation, so-called the “first-step” sign, and localized tenderness is typically elicited by palpitation on the anteromedial aspect of the plantar heel. Achilles tendinopathy is usually caused by excessive mechanical loading, such as is seen with increased running. In the setting of Achilles tendinopathy, the pain is typically achy and sharp, worsening with increased activity, and tenderness along the tendon is usually evoked by palpation. Achilles tendinopathy can be classified as mid-portion (located 2–6 cm from the calcaneal insertion) or insertional tendinopathy (located within 2 cm of the calcaneal insertion) according to the location. Achilles tendinopathy and plantar fasciitis can co-present with one another. Anatomically, the medial head of the gastrocnemius muscle fascicles demonstrates a continuation with plantar fascia in the form of periosteum in some patients [2]. Clinically, the hyperpronated foot can produce harmful mechanical stress on the plantar fascia (the tendon aponeurosis for the superficial layer of the intrinsic muscles of the foot) as well as the Achilles tendon [3]. Moreover, running-related injuries predominantly involve Achilles tendinopathy and plantar fasciitis in the foot and ankle area, supporting the potential for coexistence of both diseases [4].

Even if a specific disease can be diagnosed clinically, it is helpful for a clinician to arrive at an appropriate diagnosis when applying an imaging test with an established diagnostic value. Ultrasonography (US) is a reliable imaging method to evaluate soft tissue structures, such as muscles, tendons, aponeurosis, and ligaments. For tendinopathy, US reveals structural abnormalities, such as tendon thickening, textural heterogeneity with intra-tendinous focal hypoechoic areas, and altered vascularity [5]. There is a consensus on the role of US measurement of the thickness of the plantar fascia in establishing the diagnosis of plantar fasciitis [6,7]. A thickness of greater than 3.8 or 4.0 mm of the plantar fascia as measured by US is diagnostic of plantar fasciitis [8]. However, few studies have reported the diagnostic value of tendon thickness as measured by US in insertional Achilles tendinopathy [9,10]. Some researchers have suggested that a maximal anteroposterior diameter on conventional US of greater than 6 mm could be defined as abnormal [9,10]; however, this criterion is usually only applied to suspected cases of mid-portion Achilles tendinopathy. Overall, there has been no consensus on the diagnostic role or the diagnostic cutoff value of the Achilles tendon thickness on US, especially for insertional Achilles tendinopathy. The aim of this study was therefore to assess the diagnostic utility of US measurement of the thickness and echogenicity of the Achilles tendon for the insertional Achilles tendinopathy in patients with heel pain, and to compare the results with those of the plantar fascia for the plantar fasciitis.

## 2. Methods

This study was approved by the Institutional Ethics Review Board of Kangbuk Samsung Hospital (protocol no. KBSMC 2020-09-018-001). The requirement for informed consent waived due to the study’s retrospective design.

### 2.1. Subjects

**Figure 1:**
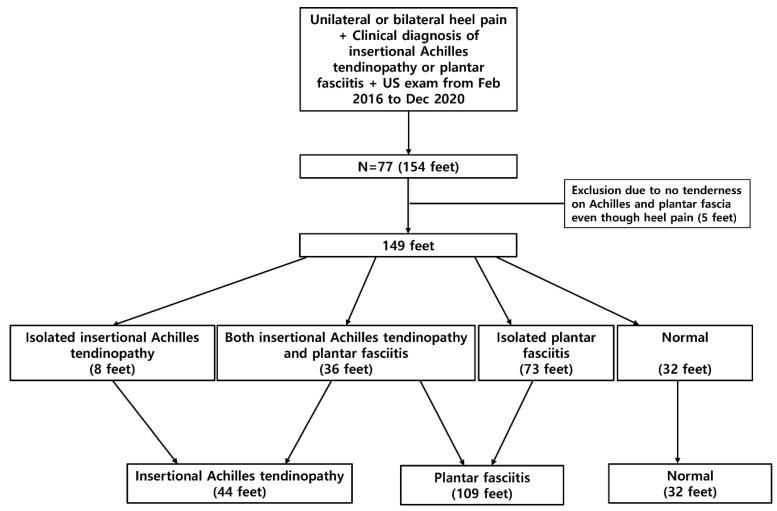
Flowchart of study population enrollment.

A retrospective chart review was performed for 77 consecutive patients with a clinical diagnosis of plantar fasciitis or insertional Achilles tendinopathy who visited at the foot clinic of Kangbuk Samsung Hospital with heel pain and underwent bilateral US examinations of the Achilles tendon and plantar fascia from February 2016 to December 2020. The clinical diagnosis of plantar fasciitis or insertional Achilles tendinopathy was established based on history-taking and physical examination by a single foot and ankle specialist with 22 years of experience in the field. Inclusion criteria for the clinical diagnosis of plantar fasciitis were chronic heel pain for more than three months, presence of the “first-step” sign, and confirmation of inferomedial calcaneal (proximal plantar fascia attachment) tenderness during physical examination. Inclusion criteria for the clinical diagnosis of insertional Achilles tendinopathy were chronic heel pain for more than three months and localized tenderness within 2 cm of the calcaneal insertion of the Achilles tendon. Normal feet that did not show heel pain and tenderness at both the inferomedial calcaneus (proximal plantar fascia attachment) and the insertion of the Achilles tendon were classified as the control group for this study. We aimed to exclude patients with recent sustained injury to the heel; systemic inflammatory diseases such as inflammatory spondyloarthropathy, rheumatoid arthritis, or gout; neuromuscular disease leading to foot deformities; history of fracture or foot surgery; infection; calcaneal apophysitis; spinal pathologies mimicking heel pain; and history of steroid injection, prolotherapy, or extracorporeal shockwave therapy. Five feet without tenderness at both the inferomedial calcaneus and the insertion of the Achilles tendon despite the presence of heel pain were excluded because they had the possibility of other conditions. Finally, 149 feet were included for the analysis of US imaging. Based on clinical diagnosis, 44 feet were diagnosed with insertional Achilles tendinopathy, 109 feet were diagnosed with plantar fasciitis, and 32 feet were classified as normal controls. Among these, 36 feet had both insertional Achilles tendinopathy and plantar fasciitis (Figure 1).

### 2.2. US and Radiographic Evaluation

**Figure 2:**
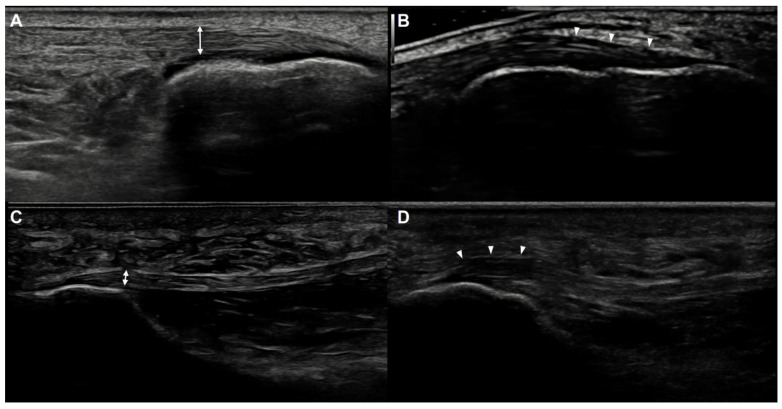
Ultrasonographic measurement of the thickness of the Achilles tendon and plantar fascia. (**A**) The maximal thickness of the insertional area of the Achilles tendon (two-sided arrow) was measured within 2 cm of the calcaneal insertion, where the tendon is attached to the bone. (**B**) A hypoechoic area and loss of the fibrillar pattern (arrowheads) were evident at the insertional area of the Achilles tendon. Note the toggling technique to keep the direction of the beam as close to perpendicular as possible in relation to the insertional area of the Achilles tendon was used. (**C**) The maximal thickness of the plantar fascia (two-sided arrow) was measured within 1 cm of the calcaneal attachment. (**D**) A hypoechoic area and loss of the fibrillar pattern (arrowheads) were evident at the attachment site of the plantar fascia.

US examinations of all patients were bilaterally performed by a single musculoskeletal specialist with 15 years of experience in musculoskeletal US. A retrospective analysis of US imaging was conducted in consensus by two musculoskeletal US specialists who were blinded to patients’ clinical diagnoses. US examinations were completed using the RS80A ultrasound system with Prestige (Samsung Medison, Co. Ltd., Seoul, Korea) equipped with a 3- to 12-MHz linear transducer. For the US evaluation of the Achilles tendon, patients were positioned lying prone with their ankles hanging free in a neutral position over the edge of the examination table. The thickness of the Achilles tendon was measured within 2 cm of the calcaneal insertion, where the tendon was attached to the bone with a maximal anteroposterior diameter in the long-axis view (Figure 2A). Echogenicity or echotexture was also assessed within 2 cm of the calcaneal insertion, where the tendon was attached to the bone. Abnormal echogenicity of the Achilles tendon was defined as hypoechoic area evident in both the longitudinal and transverse scans and heterogeneous appearance of the fibrillar pattern (Figure 2B). For the US evaluation of the plantar fascia, patients were positioned lying prone with their ankles hanging free in a slightly plantarflexed position. Plantar fascia thickness was measured as the maximal anteroposterior diameter in the long-axis view at its thickest point within 1 cm of the calcaneal attachment [6,7] (Figure 2C) and echogenicity was assessed around the medial tubercle of the calcaneus. Abnormal echogenicity of the plantar fascia was defined as hypoechoic area evident in both the longitudinal and transverse scans and heterogeneous appearance of the fibrillar pattern (Figure 2D). To confirm the difference between hypoechoic area and anisotropy, the examiner attempted to keep the direction of the beam as close to perpendicular as possible in relation to the structures in question using the techniques such as toggling the transducer and heel-to-toe rocking.

One researcher blindly viewed all weight-bearing lateral radiographs of the foot for spur grading and recorded scores for each image (i.e., none = 0, small = 1, and large = 2) [11,12]. A spur was considered large when there was a prominent peak or peak with sub-structure present, small spur being any alteration to normal surface contour of the calcaneus at the Achilles/plantar insertion. Due to their irregular shape, direct measurements of the spur length were not attempted.

### 2.3. Statistical Analysis

The Mann–Whitney *U* test was used to compare the thickness of the insertional area of the Achilles tendon or plantar fascia among the insertional Achilles tendinopathy, plantar fasciitis, and normal groups. A receiver operating characteristic curve (ROC) and the area under the ROC curve (AUC) were calculated for the thickness of the insertional Achilles tendon and plantar fascia. The chi-squared test was used to identify the difference in echogenicity and bony spur between the insertional Achilles tendinopathy group or plantar fasciitis group and the normal group. A *p*-value of less than 0.05 was considered statistically significant. Statistical analysis was performed using SPSS Statistics version 21.0 (IBM Corporation, Armonk, NY, USA).

A power analysis indicated that sample sizes of 22 patients in each group would be required to show significant differences in the thickness of Achilles tendon with a mean difference of 1.5 mm and a standard deviation of 1 mm at an α level of 0.05 and a β-value of 0.10.

## 3. Results

A total of 149 feet among the 77 study participants received US evaluation. Forty-nine patients were female (63.6%) and 28 were male (26.4%). A total of 44 feet were diagnosed with insertional Achilles tendinopathy, 109 feet were diagnosed with plantar fasciitis, and 32 feet were classified as normal controls (Figure 1). The mean age of the study participants was 51.7 ± 12.9 years, and the mean duration of symptoms was 12.7 months. The mean body mass index was 25.2 ± 3.7 kg/m^2^ with no significant differences between groups (insertional Achilles tendinopathy, 26.0 ± 4.9 kg/m^2^; plantar fasciitis, 25.0 ± 3.7 kg/m^2^; and normal, 25.8 ± 3.7 kg/m^2^).

**Figure 3:**
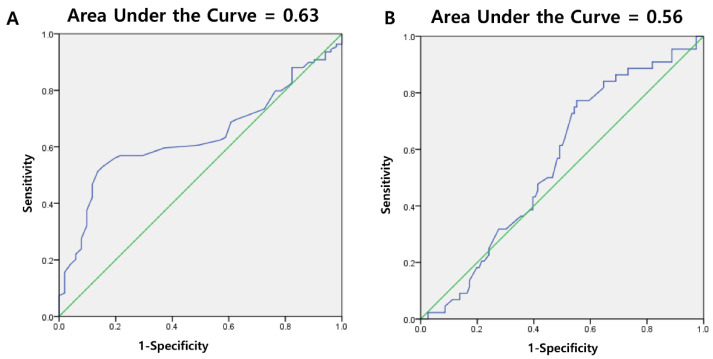
Receiver operating characteristic (ROC) curve and area under the ROC curve (AUC) for the diagnosis of plantar fasciitis (**A**) and insertional Achilles tendinopathy (**B**) using measurements of the plantar fascia and insertional Achilles tendon thickness collected using ultrasonography (AUC = 0.63 and 0.56, respectively).

The mean thickness of the plantar fascia in patients with plantar fasciitis (*n* = 109 feet) was 3.74 ± 1.17 mm, while that of normal feet (*n* = 32 feet) was 3.18 ± 0.75 mm. There was a significant difference in the thickness of the plantar fascia between the plantar fasciitis and normal groups (*p* = 0.032) (Table 1). A cutoff value of 3.8 mm for the plantar fascia thickness yielded a sensitivity of 48.6% and specificity of 90.6% (Table 2). The AUC for the thickness of the plantar fascia was 0.63 (Figure 3A). Of 109 feet with plantar fasciitis, 60 had abnormal findings of echogenicity, whereas only three among the 32 normal feet had abnormal echogenicity results. There was a significant difference in the echogenicity of the plantar fascia between the plantar fasciitis and normal groups (*p* < 0.001) (Table 1). Abnormal echogenicity yielded a sensitivity of 55.0% and specificity of 90.6%. The combination of the cutoff value of the plantar fascia thickness or abnormal echogenicity yielded a sensitivity of 56.0% and specificity of 84.4%. There was no significant difference in the frequency of bony spur on plantar fascia between the plantar fasciitis and normal groups (*p* = 0.816).

The mean thickness of the insertional area of the Achilles tendon in patients with insertional Achilles tendinopathy (*n* = 44 feet) was 4.23 ± 0.67 mm, while the same in normal feet (*n* = 32 feet) was 4.01 ± 0.86 mm. As such, there was no significant difference in the thickness of the insertional Achilles tendon between the insertional Achilles tendinopathy and normal groups (*p* = 0.132) (Table 3). The AUC for the thickness of the insertional area of the Achilles tendon was 0.56 (Figure 3B). Of the 44 feet with insertional Achilles tendinopathy, 20 had abnormal echogenicity findings, whereas there were no abnormal echogenicity results pertaining to the Achilles tendon in the normal feet. There was a significant difference in the echogenicity of the insertional area of the Achilles tendon between the insertional Achilles tendinopathy and normal groups (*p* < 0.001) (Table 3). There was no significant difference in the frequency of bony spur on insertional Achilles tendon between the insertional Achilles tendinopathy and normal groups (*p* = 0.326).

## 4. Discussion

This study aimed to evaluate the diagnostic utility of measuring the thickness of the Achilles tendon and plantar fascia by US in two representative diseases of heel pain, insertional Achilles tendinopathy and plantar fasciitis. There was a significant difference observed in the thickness of the plantar fascia between the plantar fasciitis and normal groups. However, there was no significant difference in the thickness of the insertional area of the Achilles tendon between the insertional Achilles tendinopathy and normal groups. This outcome suggests that US measurement of the thickness of the Achilles tendon might not reflect the clinical status of insertional Achilles tendinopathy, unlike that of plantar fasciitis. This finding is consistent with results of previous reports suggesting that thickness measurements on imaging do not necessarily accurately indicate the presence of Achilles tendinopathy and vice versa, while a tendon thickness of greater than 6 mm has been commonly used as a diagnostic criterion for Achilles tendinopathy [9,13,14,15,16].

There is consistent evidence of an obviously increased diameter or cross-sectional area in the cases of Achilles tendinopathy as compared with in asymptomatic controls [9,17,18,19,20,21,22]. However, most studies included patients with mid-portion Achilles tendinopathy [9,17,18,20,21,22]. For insertional Achilles tendinopathy, only a few studies have evaluated the diameter or cross-sectional area on US [14,19,23]. Chimenti et al. investigated insertional Achilles tendinopathy and reported that the side with insertional Achilles tendinopathy (6.4 ± 1.7 mm) had a larger tendon diameter than that on the healthy side (4.4 ± 0.7 mm) [14]. There is a unique structural difference that exists between the mid-portion and insertional Achilles tendon: While the mid-portion Achilles tendon principally receives consistent tensile loads parallel to the direction of the tendon fiber, the forces applied at the insertional Achilles tendon are non-uniform strains and compressive loads [14,19,24]. Such a difference in the direction of force on the mid-portion and insertional Achilles tendon influences US morphologic changes in Achilles tendinopathy. In cases of mid-portion Achilles tendinopathy, US usually reveals local tendon thickening with a difference of more than 1 mm relative to the distal Achilles tendon or diffuse tendon thickening along the mid-portion. On the other hand, it is thought that this may influence changes in the fiber array or mechanical properties rather than tendon thickening in insertional Achilles tendinopathy since not only tensile force but also the compressive load act on the insertional Achilles tendon. Therefore, this explanation seems to be in line with the results of our study, which has been shown to have more influence on the change in echotexture than tendon thickness on US in insertional Achilles tendinopathy. However, this hypothesis does not fully explain the increased fascia thickness and low echogenicity on US in patients with plantar fasciitis because the vertical fiber at the proximal attachment of plantar fascia also receives not only shear forces between the plantar subcutaneous tissue and plantar fascia but also tensile loads to limit the depression of medial longitudinal arch [25].

In terms of MRI study on the insertional Achilles tendinopathy, Solia et al. reported the size of the Achilles tendon on MRI (in the thickest anteroposterior dimension) in an asymptomatic person usually is 6 mm or less with an average size of 5.20 ± 0.73 mm on axial images, and a normal tendon is a homogeneous low-signal structure on short tau inversion recovery (STIR) imaging [26]. Karjalainen et al. showed that the level of the palpable tenderness correlated well with the level of increased signal intensity of the paratenon seen on STIR images, and all 28 patients with abnormal MR imaging findings at the level of the insertion of the Achilles tendon also had maximal pain and tenderness at that level [16]. On MRI, the Achilles tendon is thickened distally with vague, ill-defined longitudinal high signal and the enthesophyte can show evidence of marrow edema, especially in patients with acute symptoms [27]. In a comparative study with US, graded MRI appearance was associated with clinical outcome but US was not [9], and MRI revealed the highest overall diagnostic accuracy for the diagnosis of both insertional and mid-portion Achilles tendinopathy [28]. Additionally, MRI can provide extensive information about the internal morphology of the tendon and surrounding bone as well as other soft tissue, and MRI is superior to US in detecting incomplete tendon ruptures [29]. Based on these findings, if US findings remains unclear in patients with heel pain, MRI could give additional information. However, the data should be interpreted with caution, and correlated to the carful clinical assessment because many authors have also reported abnormal signals in MR images of asymptomatic tendons.

Tendinopathy is basically a clinical diagnosis for pain and dysfunction due to tendon injury [30]. Most tendon injury is caused by repetitive mechanical loading that leads to repetitive strain damage within the tendon. Microscopically, with tendon injury, negative changes in the tendon load-bearing matrix, such as increased concentrations of type III collagen, ground substance, glycosaminoglycan, and a greater number of abnormal tenocytes, begin to occur [5,31]. Then, further accumulation of mechanical loading brings about macroscopic structural changes that are detectable with US [5,31]. Typical US findings of tendinopathy include diffuse or focal tendon thickening, hyper- or hypoechogenicity, irregular fiber hypertrophy, calcification, and increased vascularity [5]. There can be an interval lag and reverse sequence between the onset of clinical symptoms of tendinopathy and the time point at which abnormal findings are observed on US. This issue can affect the diagnostic yield of US in the diagnosis of tendinopathy.

Our results demonstrated that contrary to the thickness, there was a significant difference in the echogenicity of the insertional area of the Achilles tendon between the insertional Achilles tendinopathy and normal groups. There have been few studies to evaluate the echogenicity of insertional area of the Achilles tendon. A previous study demonstrated that the echogenicity of involved side was lower than that of uninvolved side in patients with insertional Achilles tendinopathy [14]. As the Achilles tendon approaches the attachment site, the direction of the tendon fiber changes from horizontal to more vertical. This anatomic characteristic can cause an artefactual hypoechoic appearance mimicking tendinopathy. Careful attention to adjust the angle of incidence of US beam is required to assess this area correctly. Our results suggested that even though no evidence of definite increased thickness, the assessment of echogenicity would be helpful in the diagnosis of insertional Achilles tendinopathy.

A previous systematic review and meta-analysis revealed that a thickness value of the plantar fascia of more than 4.0 mm is diagnostic of plantar fasciitis [7,32]. A previous study in the Korean population suggested that a thickness of greater than 3.8 mm was a clinically meaningful US finding of plantar fasciitis [8]. In the present study, the thickness of the plantar fascia in patients with plantar fasciitis was significantly thicker than in normal controls, but the mean thickness of the plantar fascia in patients with plantar fasciitis (*n* = 109 feet) was 3.74 mm, which appears to be less thick than previously reported cases with a mean maximal thickness of 2.9 to 6.9 mm [6,7,33,34,35]. Our finding that the thickness of the plantar fascia in patients with plantar fasciitis was less than that in prior research can be attributed to the following causes. First, there may have been selection bias in our study with respect to included patients undergoing treatment that affected the thickness of the plantar fascia, even though we intended to exclude patients with a history of steroid injection, prolotherapy, or extracorporeal shockwave therapy. Second, in cases of plantar fasciitis as well, US findings, such as the thickness of the plantar fascia, did not always correlate with clinical status and vice versa. Previous studies have reported high sensitivity and specificity values of US based on a cutoff value of 4 mm for the diagnosis of plantar fasciitis [6,7]; however, there have also been contrasting results that only 42.7% to 60% of patients with plantar fasciitis exhibited thickening of the plantar fascia to more than 4 mm on US and some research suggests the mean plantar fascia thickness is 2.9 mm in cases of plantar fasciitis [35,36,37]. Our results demonstrated that a cutoff value of 3.8 mm for the plantar fascia thickness yielded a sensitivity of 48.6% and specificity of 90.6% with less accurate diagnostic value (AUC 0.63). Our results suggested that patients with plantar fasciitis might present with thickness of the plantar fascia of less than 4.0 mm on US. Even if the thickness of the plantar fascia is less than 4.0 mm on US, plantar fasciitis can be diagnosed in consideration of clinical suspicion and other pathologic US findings.

This study had several limitations. First, this study was retrospective in nature. Second, participant age, sex, height, and weight were not matched between the insertional Achilles tendinopathy or plantar fasciitis and normal groups and the thickness of the Achilles tendon can be affected uniquely by these variables [38,39]. Third, we did not assess the inter-observer or intra-observer reliability of measuring the thickness and echogenicity of the Achilles tendon and plantar fascia. Fourth, the thickness of the Achilles tendon was measured in the long-axis plane, which may lead to overestimation of its thickness due to the tendon’s oblique course. Fifth, the anisotropy artefact of the tendon fiber in the fibrocartilage attachment zone can mimic the low echogenicity of insertional area of Achilles tendon even though the examiner attempted to keep the direction of the beam as close to perpendicular as possible in relation to the anatomy in question.

## 5. Conclusions

In conclusion, US measurement of the Achilles tendon thickness appears to have a limited role in the diagnosis of insertional Achilles tendinopathy, unlike in mid-portion Achilles tendinopathy or plantar fasciitis. Therefore, additional measurement of other US parameters, such as echogenicity, vascularity, or stiffness, would be needed in the US diagnosis of insertional Achilles tendinopathy.

## Figures and Tables

**Table 1:** Comparisons of ultrasonographic findings between the plantar fasciitis and normal groups.

	Plantar Fasciitis (*n* = 109)	Normal (*n* = 32)	*p*-Value
Thickness (mm)	3.74 ± 1.17	3.18 ± 0.75	0.032
Abnormal echogenicity (*n*)	60	2	<0.001

**Table 2:** Diagnostic accuracy according to cutoff values of the thickness of the plantar fascia.

Cutoff Value (mm)	Sensitivity	Specificity	Youden’s Index
3.65	53.2%	84.4%	0.376
3.75	51.4%	87.5%	0.389
3.80	48.6%	90.6%	0.392
4.00	37.6%	90.6%	0.282

**Table 3:** Comparisons of ultrasonographic findings between the insertional Achilles tendinopathy and normal groups.

	Insertional Achilles Tendinopathy (*n* = 44)	Normal (*n* = 32)	*p*-Value
Thickness (mm)	4.23 ± 0.67	4.01 ± 0.86	0.132
Abnormal echogenicity (*n*)	20	0	<0.001

## Data Availability

The data presented in this study are available on request from the corresponding author.

## References

[1] Tu P. (2018). Heel Pain: Diagnosis and Management. Am. Fam. Physician.

[2] Mahan J., Damodar D., Trapana E., Barnhill S., Nuno A.U., Smyth N.A., Aiyer A., Jose J. (2020). Achilles tendon complex: The anatomy of its insertional footprint on the calcaneus and clinical implications. J. Orthop..

[3] Maffulli N., Kader D. (2002). Tendinopathy of tendo achillis. J. Bone Jt. Surg. Br..

[4] Ramskov D., Rasmussen S., Sørensen H., Parner E.T., Lind M., Nielsen R. (2018). Progression in Running Intensity or Running Volume and the Development of Specific Injuries in Recreational Runners: Run Clever, a Randomized Trial Using Competing Risks. J. Orthop. Sports Phys. Ther..

[5] Zabrzyński J., Paczesny Ł., Zabrzyńska A., Grzanka D., Łapaj Ł. (2018). Sonography in the instability of the long head of the biceps tendon confronted with histopathologic and arthroscopic findings. Folia Morphol..

[6] Aggarwal P., Jirankali V., Garg S.K. (2020). Evaluation of plantar fascia using high-resolution ultrasonography in clinically diagnosed cases of plantar fasciitis. Pol. J. Radiol..

[7] McMillan A.M., Landorf K.B., Barrett J.T., Menz H.B., Bird A.R. (2009). Diagnostic imaging for chronic plantar heel pain: A systematic review and meta-analysis. J. Foot Ankle Res..

[8] Yoon K., Kim S.B., Park J.S. (2002). Ultrasonographic Findings in Plantar Fasciitis. J. Korean Acad. Rehabil. Med..

[9] Khan K.M., Forster B.B., Robinson J., Cheong Y., Louis L., Maclean L., Taunton J.E. (2003). Are ultrasound and magnetic resonance imaging of value in assessment of Achilles tendon disorders? A two year prospective study. Br. J. Sports Med..

[10] Richards P.J., Dheer A.K., McCall I.M. (2001). Achilles tendon (TA) size and power Doppler ultrasound (PD) changes compared to MRI: A preliminary observational study. Clin. Radiol..

[11] Toumi H., Davies R., Mazor M., Coursier R., Best T.M., Jennane R., Lespessailles E. (2014). Changes in prevalence of calcaneal spurs in men & women: A random population from a trauma clinic. BMC Musculoskelet Disord..

[12] Usuelli F.G., Di Silvestri C.A., D’Ambrosi R., Maccario C., Tan E.W. (2018). Return to sport activities after medial displacement calcaneal osteotomy and flexor digitorum longus transfer. Knee Surg. Sports Traumatol. Arthrosc..

[13] Bakkegaard M., Johannsen F.E., Højgaard B., Langberg H. (2015). Ultrasonography as a prognostic and objective parameter in Achilles tendinopathy: A prospective observational study. Eur. J. Radiol..

[14] Chimenti R.L., Flemister A.S., Tome J., McMahon J.M., Flannery M.A., Xue Y., Houck J.R. (2014). Altered tendon characteristics and mechanical properties associated with insertional achilles tendinopathy. J. Orthop. Sports Phys. Ther..

[15] De Jonge S., Tol J.L., Weir A., Waarsing J.H., Verhaar J.A., de Vos R.J. (2015). The Tendon Structure Returns to Asymptomatic Values in Nonoperatively Treated Achilles Tendinopathy but Is Not Associated with Symptoms: A Prospective Study. Am. J. Sports Med..

[16] Karjalainen P.T., Soila K., Aronen H.J., Pihlajamäki H.K., Tynninen O., Paavonen T., Tirman P.F. (2000). MR imaging of overuse injuries of the Achilles tendon. AJR Am. J. Roentgenol..

[17] Arya S., Kulig K. (2010). Tendinopathy alters mechanical and material properties of the Achilles tendon. J. Appl. Physiol..

[18] Child S., Bryant A.L., Clark R.A., Crossley K.M. (2010). Mechanical properties of the achilles tendon aponeurosis are altered in athletes with achilles tendinopathy. Am. J. Sports Med..

[19] Chimenti R.L., Cychosz C.C., Hall M.M., Phisitkul P. (2017). Current Concepts Review Update: Insertional Achilles Tendinopathy. Foot Ankle Int..

[20] Kulig K., Chang Y.J., Winiarski S., Bashford G.R. (2016). Ultrasound-Based Tendon Micromorphology Predicts Mechanical Characteristics of Degenerated Tendons. Ultrasound Med. Biol..

[21] Ooi C.C., Schneider M.E., Malliaras P., Chadwick M., Connell D.A. (2015). Diagnostic performance of axial-strain sonoelastography in confirming clinically diagnosed achilles tendinopathy: Comparison with B-mode ultrasound and color Doppler imaging. Ultrasound Med. Biol..

[22] Wang H.K., Lin K.H., Su S.C., Shih T.T., Huang Y.C. (2012). Effects of tendon viscoelasticity in achilles tendinosis on explosive performance and clinical severity in athletes. Scand. J. Med. Sci. Sports.

[23] Nicholson C.W., Berlet G.C., Lee T.H. (2007). Prediction of the success of nonoperative treatment of insertional achilles tendinosis based on MRI. Foot Ankle Int..

[24] Obst S.J., Heales L.J., Schrader B.L., Davis S.A., Dodd K.A., Holzberger C.J., Beavis L.B., Barrett R.S. (2018). Are the Mechanical or Material Properties of the Achilles and Patellar Tendons Altered in Tendinopathy? A Systematic Review with Meta-analysis. Sports Med..

[25] Christie S., Styn G., Ford G., Terryberry K. (2019). Proximal Plantar Intrinsic Tendinopathy: Anatomical and Biomechanical Considerations in Plantar Heel Pain. J. Am. Podiatr. Med. Assoc..

[26] Soila K., Karjalainen P.T., Aronen H.J., Pihlajamäki H.K., Tirman P.J. (1999). High-resolution MR imaging of the asymptomatic Achilles tendon: New observations. AJR Am. J. Roentgenol..

[27] Pierre-Jerome C., Moncayo V., Terk M.R. (2010). MRI of the Achilles tendon: A comprehensive review of the anatomy, biomechanics, and imaging of overuse tendinopathies. Acta Radiol..

[28] Gatz M., Bode D., Betsch M., Quack V., Tingart M., Kuhl C., Schrading S., Dirrichs T. (2021). Multimodal Ultrasound Versus MRI for the Diagnosis and Monitoring of Achilles Tendinopathy: A Prospective Longitudinal Study. Orthop. J. Sports Med..

[29] Maffulli N., Longo U.G., Kadakia A., Spiezia F. (2020). Achilles tendinopathy. Foot Ankle Surg..

[30] Khan K.M., Cook J.L., Kannus P., Maffulli N., Bonar S.F. (2002). Time to abandon the “tendinitis” myth. BMJ.

[31] Zabrzyński J., Gagat M., Paczesny Ł., Łapaj Ł., Grzanka D. (2018). Electron microscope study of the advanced tendinopathy process of the long head of the biceps brachii tendon treated arthroscopically. Folia Morphol..

[32] Turan A., Tufan A., Mercan R., Teber M.A., Tezcan M.E., Bitik B., Goker B., Haznedaroğlu S. (2013). Real-time sonoelastography of Achilles tendon in patients with ankylosing spondylitis. Skeletal. Radiol..

[33] Ermutlu C., Aksakal M., Gümüştaş A., Özkaya G., Kovalak E., Özkan Y. (2018). Thickness of plantar fascia is not predictive of functional outcome in plantar fasciitis treatment. Acta Orthop. Traumatol. Turc..

[34] Hansen L., Krogh T.P., Ellingsen T., Bolvig L., Fredberg U. (2018). Long-Term Prognosis of Plantar Fasciitis: A 5- to 15-Year Follow-up Study of 174 Patients with Ultrasound Examination. Orthop. J. Sports Med..

[35] Ozdemir H., Yilmaz E., Murat A., Karakurt L., Poyraz A.K., Ogur E. (2005). Sonographic evaluation of plantar fasciitis and relation to body mass index. Eur. J. Radiol..

[36] Park J.W., Yoon K., Chun K.S., Lee J.Y., Park H.J., Lee S.Y., Lee Y.T. (2014). Long-term outcome of low-energy extracorporeal shock wave therapy for plantar fasciitis: Comparative analysis according to ultrasonographic findings. Ann. Rehabil. Med..

[37] Sabir N., Demirlenk S., Yagci B., Karabulut N., Cubukcu S. (2005). Clinical utility of sonography in diagnosing plantar fasciitis. J. Ultrasound Med..

[38] Pang B.S., Ying M. (2006). Sonographic measurement of achilles tendons in asymptomatic subjects: Variation with age, body height, and dominance of ankle. J. Ultrasound Med..

[39] Qahtani M.A., Mirza E.H. (2016). Thickness of Achilles Tendon is BMI Dependant. J. Coll. Physicians Surg. Pak..

